# MRI Visualization of CSF Flow

**DOI:** 10.1186/2045-8118-12-S1-P33

**Published:** 2015-09-18

**Authors:** Edward Frederick Melamed, Skorn Ponrartana, Eisha Anne Christian, Matthew Borzage, Stefan Bluml, J Gordon McComb

**Affiliations:** 1 Division of Neurosurgery, Children's Hospital Los Angeles, CA, USA; 2 Department of Radiology, Children's Hospital Los Angeles, CA, USA; 3 Department of Neurological Surgery, Keck School of Medicine, University of Southern California, Los Angeles, CA, USA

## Introduction

The ability to non-invasively establish patency of cerebral spinal fluid (CSF) flow between adjacent central nervous system (CNS) compartments is of importance in the diagnosis and treatment of patients with various areas of CSF flow obstruction. Recent advances in magnetic resonance imaging (MRI) technology allows for real-time magnetic labeling of CSF to directly visualize flow through different compartments.

## Methods

The presence of CSF flow was examined at the aqueduct of Sylvius (AS), the foramen of Monro (FM), the floor of the third ventricle (3rd V), and the foramen magnum (FMag) using a modification of arterial spin labeling (ASL). The studies were compared with clinical information and classified as true positive, true negative, false positive, and false negative based on expectation of patency.

## Results

A total of 68 flow studies were done on 44 patients. High correlation with true positive was seen at all four sites. No flow was visualized in 25 readings of the AS; in 9 of those cases we expected flow to be present. Table 1.

**Table 1:** 

AS	FM	3rd	V	FMag
Positive-True	26/26	8/8	12/12	30/30

False	0/26	0/8	0/12	0/30

Negative-True	16/25	0/2	2/3	1/3

False	9/25	2/2	1/3	2/3

Specificity	100%	0%	100%	100%

Sensitivity	74%	80%	92%	94%

Negative	LR 0.26	Undefined	0.08	0.06

## Conclusion

Establishing qualitative patency between adjacent CSF compartments using MRI is possible with a modified ASL technique. This technique has excellent (above 80%) sensitivity, specificity, and negative likelihood ratio in three out of the four regions studied.

## References

[B1] Christian EA Melamed EF Peck E Krieger MD McComb JG Surgical Management of Hydrocephalus Secondary to Intraventricular Hemorrhage in the Preterm Infant J Neurosurg Pediatrics [Accepted for Publication June 18, 2015] 10.3171/2015.6.PEDS15132 26565942

